# The relationship between belief in a just world and learning burnout in Chinese junior high school students: a moderated mediation model

**DOI:** 10.3389/fpsyg.2025.1650231

**Published:** 2025-10-03

**Authors:** Xiaodi Wang, Xuefeng Wei, Xue Zhang, Yuying Zou, Kai Zhang

**Affiliations:** ^1^College of Education, Ludong University, Yantai, Shandong Province, China; ^2^Institute for Education and Treatment of Problematic Youth, Ludong University, Yantai, Shandong Province, China

**Keywords:** belief in a just world, learning burnout, perceived discrimination, self-efficacy, junior high school students

## Abstract

We proposed a moderated mediation model to investigate the relationship between belief in a just world and learning burnout (LB) in junior high school students, as well as the mediating effect of perceived discrimination and the moderating effect of self-efficacy. A total of 797 students (399 boys and 398 girls; age ranged from 11 to 15 years old, mean age was 13.3, SD was 1.61) from Shandong province, China, completed the Belief in a Just World Scale, Questionnaire of Perceived Discrimination, General Self-Efficacy Scale, and Learning Burnout Scale. SPSS 23.0 and PROCESS macro 4.1 (Model 7) were used to test the moderated mediation model. The findings of this study proved that belief in a just world was significantly and negatively related to LB, and perceived discrimination played a mediating role between them. Self-efficacy moderated the mediating path, the effect being weaker for students with a high level of self-efficacy. These findings demonstrated that perceived discrimination is the critical link between belief in a just world and LB, and high self-efficacy helps prevent perceived discrimination and LB.

## 1 Introduction

As a Chinese saying goes, “good is rewarded with good, and evil with evil.” Belief in a just world (BJW) reflects individuals’ perception that the world operates fairly (Lerner and Simmons, 1966). Formed through developmental processes, it serves as a personal trait influencing justice cognitions (Faccenda and Pantaléon, 2011). With growing recognition in positive psychology, BJW’s adaptive functions—particularly in mitigating negative emotions—have gained empirical support (Dalbert, 1999; Song, 2020; Zhou and Guo, 2013).

Learning burnout (LB) was one of the common negative learning psychology of students (Lian et al., 2005), which will not only harm students’ physical and mental health and affect their social interactions (Demerouti et al., 2001; Guo and Zhou, 2008) but also positively predict their work burnout (Robins et al., 2018). Concurrently, LB represents a prevalent issue among Chinese middle school students (Yang et al., 2024).

Previous researchers have proposed applying positive psychology to the study of LB, focusing on the supporting role of individual positive and potential strengths and qualities in alleviating LB (Shen et al., 2019). However, compared with psychological capital, core self-evaluation, and other variables, the research on the influence of BJW, which is also one of the positive psychological resources, on LB of junior high school students is relatively scarce (Gan et al., 2007; Song and Duan, 2014).

Due to the limited social experience, junior high school students have problems such as immature thoughts and impulsive and bold behavior (Xu et al., 2017). Junior high school students exhibit heightened vulnerability during social transitions in China. Their developing cognition may amplify perceptions of injustice, potentially escalating LB.

In conclusion, current studies predominantly test single mediators (e.g., self-esteem) or moderators (e.g., social support) in isolation. Crucially, perceived discrimination (PD)—a subjective sense of unfair treatment due to group membership (Brownfield, 2005)—is overlooked as a mediator linking BJW to LB. Moreover, self-efficacy’s moderating role in this pathway is unexamined despite its established buffering effect in stress coping (Schwarzer et al., 1999). This study bridges these gaps by proposing a moderated mediation model wherein BJW reduces LB via decreased PD, with self-efficacy weakening this indirect path.

Junior high students in China face intensified academic competition and educational inequities (e.g., streaming by achievement), heightening PD risks across intelligence, appearance, and socioeconomic dimensions. Cultural values (e.g., Confucian collectivism) further amplify self-efficacy’s protective function.

Thus, the present study aimed to construct and empirically test a moderated mediation model to elucidate the relationship between BJW and LB among Chinese junior high school students. Specifically, we proposed that PD mediates the negative effect of BJW on LB, with self-efficacy attenuating this mediating pathway. This investigation offers the first comprehensive examination of this psychological mechanism in adolescents, thereby providing theoretical insights and practical strategies for mitigating LB through targeted interventions.

## 2 Literature review and research hypotheses

### 2.1 Belief in a just world and learning burnout

LB arises from chronic learning stress that cannot be properly dealt with (Liu, 2012). It refers to the state of emotional, behavioral, and cognitive exhaustion caused by excessive learning pressure or lack of interest in learning, usually manifested as decreased self-efficacy, physiological exhaustion, emotional exhaustion, and deindividuation (Meier and Schmeck, 1985; Tuominen and Salmela, 2014). External environmental factors (academic, life pressure, and social support) and individual factors (self-efficacy and coping style) affected students’ LB (Alarcon et al., 2011). Among them, personal factors are most closely related to students’ LB (Gong, 2010).

The stress coping model (SCM), based on BJW, proposed that BJW, as a psychological resource, can help individuals evaluate and adapt to stressful events and can help individuals carry out positive cognitive assessment when coping with stressful events (Tomaka and Blascovich, 1994). The Effort–Reward Model (ERM), which is used to solve burnout formation, believes that burnout occurs when people feel that the amount of effort they are putting in exceeds the amount they are getting in return, and that the situation cannot be effectively addressed in the short term (Siegrist, 1996). When individuals’ BJW is at a low level, that is, they do not think that efforts will be rewarded accordingly, they may show burnout psychology in their learning process. A previous study also found that high school students’ BJW can negatively predict their LB (Song, 2020). Therefore, we predict that junior high school students with stronger BJW will demonstrate lower susceptibility to LB.

### 2.2 The mediating role of perceived discrimination

As a subjective experience, perception of discrimination (PD) is an individual’s perception of differential treatment or unfair treatment due to the membership of the group to which he belongs, which often causes a series of negative effects on his psychological adaptation, including the inability to correctly understand and evaluate himself and the decrease of self-efficacy (Brownfield, 2005; Liu et al., 2011; Zhang et al., 2016). Previous research has confirmed a significant negative relationship between BJW and PD (Zhang et al., 2022). According to the ecosystem theory, adolescent psychology and behavior are influenced by dynamic person–environment interactions across microsystem (Wang and Meng, 2015). The influence of BJW on PD, in addition to the discrimination behavior in the objective environment, is also inseparable from the influence of individual factors (Yang and Zhang, 2018). BJW can help people re-establish a sense of justice on a cognitive level and reduce the likelihood of perceived discrimination (Wang and Meng, 2015). At the same time, as a personal resource, BJW also helps to reduce the negative impact of injustice, maintain mental health, and motivate individuals to focus on long-term goals (Song, 2020). Previous studies have proved that BJW has a significant negative effect on PD, and individuals with a higher level of BJW report fewer cases of discrimination (Song et al., 2018).

Existing studies have shown that PD has a direct impact on LB. The changes of contemporary social values, family life form and structure, and social culture have a certain degree of negative impact on junior high school students’ mental health and learning psychology (Chen, 2016). Compared with objective discrimination, subjective PD has a greater impact on the psychological health of junior high school students (Liu et al., 2011). PD of junior high school students is mainly reflected in their subjective perception of being differentiated or unfairly treated when they face the uncertain world, and PD will further have a negative impact on their academic development (Xu et al., 2017). The influence of LB is mainly due to the support and feedback of the external environment, PD, as the pressure source from an external environment in the development of the individual body and mind was significantly positively related to LB (Dong et al., 2020). BJW, as a personal resource, can predict PD and thus LB, therefore, PD may be the key link between BJW and LB. Therefore, after a systematic review of the existing evidence, we hypothesize that PD may mediate the association between BJW and LB, wherein higher BJW reduces PD, which in turn mitigates LB risk.

### 2.3 The moderating role of self-efficacy

Self-efficacy includes people’s belief in their own ability and their sense of control over their behavior (Schwarzer et al., 1999). According to social cognitive theory, if people believe that the world is unjust (they will not succeed even if they put in the effort), they may show self-demeaning tendencies and a decline in self-efficacy, and finally appear to avoid performing tasks (Locke, 1987). When manifested in junior high school students, LB may appear. Modified labeling theory suggests that when a person was classified into a particular group, their perception of social discrimination will increase, they become more sensitive, and they are more likely to engage in defensive responses such as avoidance, burnout, and rejection (Link et al., 1989) Discrimination, as a negative evaluative feedback, was directly related to individual self-awareness and evaluation, and low level of self-efficacy will expand the negative effect of PD (Brownfield, 2005). The learned helplessness theory proves that the decrease of individual self-efficacy will expand the level of PD, and thus increase the sense of an uncontrollable environment (Cao et al., 2024). As learned helplessness intensifies, individuals will identify with certain social biases and impressions, resulting in a negative self-concept and low self-evaluation, which in turn leads to self-doubt, resulting in a low level of personal accomplishment (Corrigan et al., 2009). This low intensity of personal achievement, in the group of junior high school students, manifests as LB. On the contrary, a higher level of self-efficacy will enable individuals to conduct self-awareness from a positive perspective and choose a more reasonable attitude and correct treatment formula to respond to differences, thus showing a higher motivation for learning achievement (Konaszewski et al., 2021). Previous studies have determined that there is a direct correlation between self-efficacy and LB, which can negatively predict LB. Therefore, we hypothesize that self-efficacy negatively moderates the indirect effects between BJW and LB via PD.

### 2.4 The present research hypotheses

**Figure 1:**
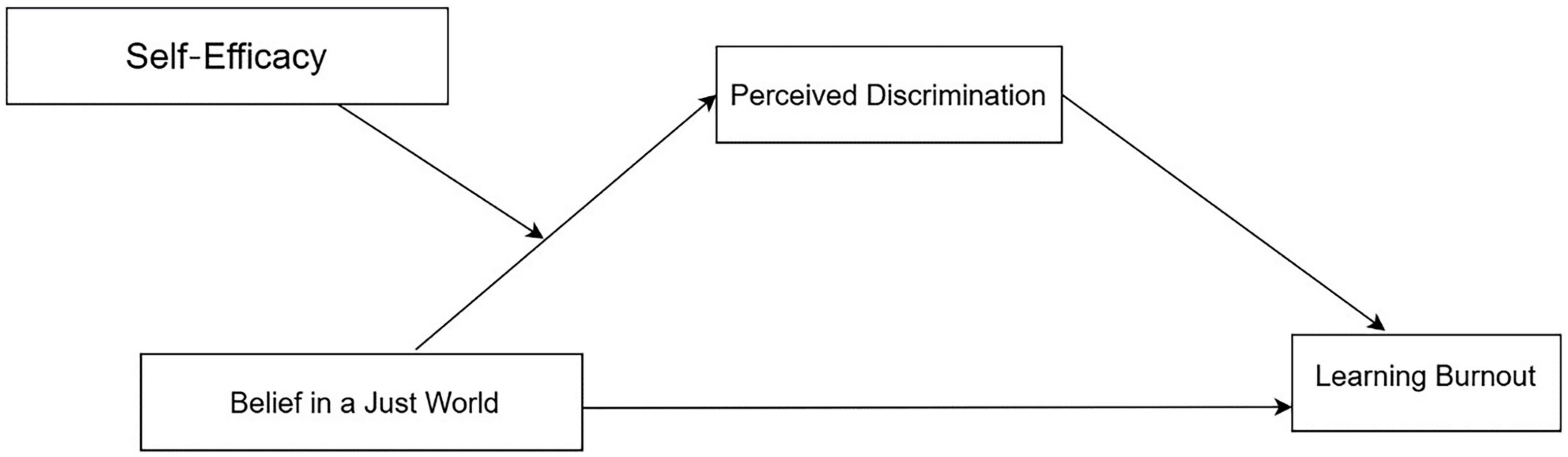
Hypothesized model.

LB is closely related to the internal and external problems of junior high school students (Gomez et al., 2022; Salmela-Aro et al., 2009). This study investigates the psychological mechanisms of LB among Chinese junior high school students and aims to inform targeted interventions that simultaneously enhance academic outcomes and mental health in adolescence. To construct a moderated mediation model with BJW as the independent variable and LB as the dependent variable, we examine: (1) how BJW operates as a protective factor against LB through reduced PD, and (2) whether self-efficacy amplifies this protective pathway (see Figure 1). Specifically, we hypothesize that:

*Hypothesis 1*: Higher BJW will predict lower LB indirectly through reduced PD.

*Hypothesis 2*: The protective effect of BJW against PD would be stronger for adolescents with high self-efficacy.

## 3 Materials and methods

### 3.1 Participants and procedure

A total of 830 students from four junior high schools in Shandong province, China were selected as research participants, 830 questionnaires were distributed, and 797 valid questionnaires were obtained (return rate was 96%). Participants’ mean age was 13.3 (SD was 1.61, age ranged from 11 to 15 years old); here 399 (50.06%) were boys and 398 (49.94%) were girls; the growth environment was 307 (38.52%) urban and 490 (61.48%) rural; and 135 (16.94%) were the only children and 662 (83.06%) were non-only children.

The four junior high schools were purposively selected from Yantai city, Shandong Province, to ensure geographical and socioeconomic diversity. Specifically, two schools were located in urban districts and two in rural counties. School selection criteria included: (1) representative socioeconomic backgrounds: Urban schools served communities with mid-to-high parental education levels and diverse occupations; rural schools primarily served agricultural households. (2) Varied resource profiles: All schools met provincial standards for teacher–student ratios and infrastructure, but differed in funding levels. This approach captured key dimensions of educational diversity in Shandong. While the sample may not generalize to all Chinese regions, it reflects socioeconomic gradients and institutional characteristics common in Eastern China’s public education system.

First, the ethics review committee of Ludong University approved the study. Second, we obtained the informed consent of the participants’ parents. The students participated in the study anonymously. Third, our research obtained the permission of the school principal and the help of the school teachers. In the computer class, we distributed the electronic questionnaire through the network platform of the Questionnaire Star. Fourth, professional psychology graduate students conducted the survey and were responsible for interpreting when participants had questions about the questionnaire. Finally, we gave the participants a notebook as a reward for participating.

### 3.2 Measures

#### 3.2.1 Belief in a just world

A revised Chinese version of the Belief in a Just World Scale (BJWS) (Du et al., 2007), developed by Dalbert (1999), was used. BJWS, with a total of 13 items, included two dimensions: general just world belief (six items) and individual just world belief (seven items). A sample item of general just world belief was “I am convinced that justice always triumphs over injustice.” A sample item of individual just world belief was “I believe I usually get what I deserve.” Responses are rated on a 5-point Likert scale ranging from 1 (totally disagree) to 5 (totally agree); higher scores indicated the higher overall level of BJW. In this study, Cronbach’s *α* was 0.95.

#### 3.2.2 Perceived discrimination

Perceived discrimination was measured by the Questionnaire of Perceived Discrimination (QPD) (Shen et al., 2009). The questionnaire has a total of 15 items and contains four dimensions: intelligence discrimination, body trait discrimination, verbal communication discrimination, and family background discrimination. Among them, the intelligence discrimination includes four items, such as “I think teachers like smart students”; the body trait discrimination includes four items, such as “My classmates gave me nicknames based on my appearance”; the verbal communication discrimination includes four items, such as “My classmates were not interested in what I said”; the family background discrimination includes three items, such as “I think students from good families do not like to play with me.” Responses are rated on a 5-point Likert scale ranging from 1 (totally disagree) to 5 (totally agree). This study used the total score as the observed score for perceived discrimination, and higher scores indicated a higher overall level of PD. In this study, Cronbach’s α was 0.95.

#### 3.2.3 Self-efficacy

The study used the general self-efficacy scale (GSES) to measure self-efficacy (Schwarzer et al., 1999). The scale included 10 items. A sample item was “When faced with a difficult problem, I can usually find several solutions.” Responses are rated on the Likert five-point scale, ranging from 1 (totally disagree) to 5 (totally agree). Higher scores indicated higher levels of self-efficacy. In this study, Cronbach’s α was 0.94.

#### 3.2.4 Learning burnout

LB was measured using the Learning Burnout Scale (LBS) (Hu and Dai, 2007). The scale has a total of 14 items and contains four dimensions: sense of learning inefficiency, feeling down, physiological exhaustion, and academic alienation. Among them, the sense of learning inefficiency includes four items, such as “I do not think I have the ability to get good grades”; the feeling down includes three items, such as “I feel very confused recently, do not know what to do”; the physiological exhaustion includes three items, such as “I get a headache when I learn for a while”; the academic alienation includes four items, such as “I want to give up learning.” Responses are rated on a 5-point Likert scale ranging from 1 (totally disagree) to 5 (totally agree). This study used the total score as the observed score for LB, and higher scores indicated a higher overall level of LB.

### 3.3 Data analysis

First, SPSS 23.0 software was used to organize and analyze the data. The missing values were replaced with means. At the same time, the data in this study were derived from participants’ self-reports, so there may be common methodological bias. To this end, we have adopted some appropriate means in the research process, such as anonymous answers, reverse scoring, etc. In addition, Harman’s single-factor test method (Podsakoff et al., 2003) was used to conduct an exploratory factor analysis of 52 items in four scales. The results showed that there were seven factors with feature roots greater than 1, and the variance explained by the first factor was 18.35%, which was lower than the evaluation standard of 40%. Therefore, no common methodological bias was detected in the data of this study.

Second, SPSS 23.0 was used for descriptive statistics and correlation analysis of each variable’s data. Third, we used the PROCESS macro 4.1 (Hayes, 2014) (Model 7) to test the moderated mediation model. The age and gender variables were controlled, and the mediated model was tested based on Bootstrap analysis. If the confidence intervals (CI) were not contained within 0, the effect was significant. Finally, we performed a simple slope test to explain the size of the moderating effect.

## 4 Results

### 4.1 Descriptive statistics

The results of descriptive statistics and relevant analysis are shown in Table 1. There was a pairwise positive correlation between all variables (*p* < 0.01), which was consistent with the hypothesis proposed in this study. In detail, BJW was significantly and negatively related to LB (*r* = −0.19) and PD (*r* = −0.17). PD was positively related to LB (*r* = 0.62) and negatively related to self-efficacy (*r* = −0.11). Self-efficacy was negatively related to LB (*r* = −0.24) and positively related to BJW (*r* = 0.49). Our study found that BJW, PD, and self-efficacy are directly related to LB, and all have an impact on LB.

**Table 1:** Descriptive statistics and correlation coefficients of each variable (*N* = 797).

Predictors	*M*	*SD*	1	2	3	4
BJW	3.89	0.84	1			
PD	1.99	0.95	−0.17^**^	1		
Self-efficacy	3.62	0.86	0.49^**^	−0.11^**^	1	
LB	2.11	0.94	−0.19^**^	0.62^**^	−0.24^**^	1

BJW, belief in a just world; PD, perceived discrimination; LB, learning burnout; ^**^p < 0.01.

To address potential demographic variations, we conducted post-hoc analyses on gender and urban–rural differences using independent samples *t*-tests (Table 2). The results showed that there was a significant difference in PD between boys and girls (*t* = −3.06, *p* < 0.01), and the PD level of girls was significantly higher than that of boys. There was a significant difference in PD between students living in urban and rural areas (*t* = −2.25, *p* < 0.05), and the PD level of students living in rural areas was significantly higher than that of students living in urban areas.

**Table 2:** Exploratory analysis of group differences (*N* = 797).

Predictors	Male	Female	*t*	*p*	*df*
BJW	3.91 ± 0.89	3.88 ± 0.78	0.54	0.59	795
PD	2.10 ± 1.05	1.90 ± 0.82	−3.06	0.002^**^	753.31
Self-Efficacy	3.68 ± 0.90	3.56 ± 0.81	1.88	0.06	795
LB	2.15 ± 1.03	2.05 ± 0.84	1.56	0.119	766.20

Predictors	Urban	Rural	*t*	*p*	*df*
BJW	3.89 ± 0.86	3.90 ± 0.82	−0.18	0.86	795
PD	1.91 ± 0.84	2.06 ± 1.01	−2.25	0.0258^*^	731.33
Self-efficacy	3.62 ± 0.84	3.62 ± 0.87	0.03	0.97	795
LB	2.06 ± 0.89	2.13 ± 0.97	−0.89	0.37	795

BJW, belief in a just world; PD, perceived discrimination; LB, learning burnout; ^**^p < 0.01, ^*^p < 0.05.

### 4.2 Test for mediating effect

First, Model 7 was used to test the mediating role of PD between BJW and LB. The results are shown in Tables 3, 4. The results of regression analysis showed that after controlling for age and gender, BJW was negatively related to LB (*β* = −0.10, *t* = −3.10, *p* < 0.01) and negatively related to PD (*β* = −0.20, *t* = −4.30, *p* < 0.001), and PD was positively related to LB (*β* = 0.60, *t* = 21.67, *p* < 0.001). Further using the deviation-corrected percentile Bootstrap method, it was found that PD had a significant mediating effect between BJW and LB, a*b = −0.12, *t* = 5.48, SE = 0.03, and 95% CI was [−0.17, −0.05]. The total effect of BJW on LB was −0.22, and the proportion of the mediating effect was 55%. Therefore, according to this finding, Hypothesis 1 was supported.

**Table 3:** Test for mediating effect between BJW and LB (*N* = 797).

Predictors	Model 1 LB		Model 2 PD		Model 3 LB	
	*β*	*t*	*β*	*t*	*β*	*t*
Age	0.09	3.18^**^	0.01	0.29	0.09	2.71^**^
Gender	0.01	0.27	−0.23	−3.544^***^	−0.11	−1.74
BJW	−0.10	−3.10^**^	−0.20	−4.30^***^	−0.21	−5.48^***^
PD					0.60	21.67^***^
*R^2^*	0.40		0.05		0.05	
*F*	133.80^***^		9.09^***^		13.81^***^	

BJW, belief in a just world; PD, perceived discrimination; LB, learning burnout; ^**^p < 0.01, ^***^p < 0.001.

**Table 4:** Test of the mediation effect between BJW and LB.

Type	*β*	*SE*	95% *CI*	REV
Indirect effect	−0.12	0.03	[−0.17, −0.05]	55%
Direct effect	−0.10	0.03	[−0.16, −0.04]	45%
Total effect	−0.22	0.39	[−0.29, −0.14]	

Total effect, direct effect and indirect effect. SE, standard error; CI, confidence interval; REV, relative effect value.

### 4.3 Test for moderated mediation

As noted in Hypothesis 2, self-efficacy would moderate the relationship between BJW and LB via PD. Then, Model 7 compiled by Hayes (the moderating variable moderates the first half path in the mediation model) was used to test the moderated mediation. After controlling for age and gender, the results showed (see Table 5) that the interaction terms of BJW and self-efficacy were negatively related to PD (*β* = −0.11, *t* = −3.25, *p* < 0.01). This suggests that the process of BJW influencing LB via PD was moderated by self-efficacy (*β* = −0.11, *t* = −3.25, *p* < 0.01).

**Table 5:** Testing for moderated mediation.

Predictors	PD		LB	
	*β*	*t*	*β*	*t*
Age	0.01	0.28	0.09	3.20
Gender	−0.23	−3.54	0.01	0.27
BJW	−0.19	−4.31^***^	−0.09	−3.1^**^
PD			0.60	21.67^***^
Self-efficacy	−0.04	−0.98^**^		
BJW*Self-efficacy	−0.11	−3.25^**^		
*R^2^*	0.05		0.40	
*F*	9.09^***^		133.80^***^	

BJW, belief in a just world; PD, perceived discrimination; LB, learning burnout; ^*^p < 0.05, ^***^p < 0.001.

**Figure 2:**
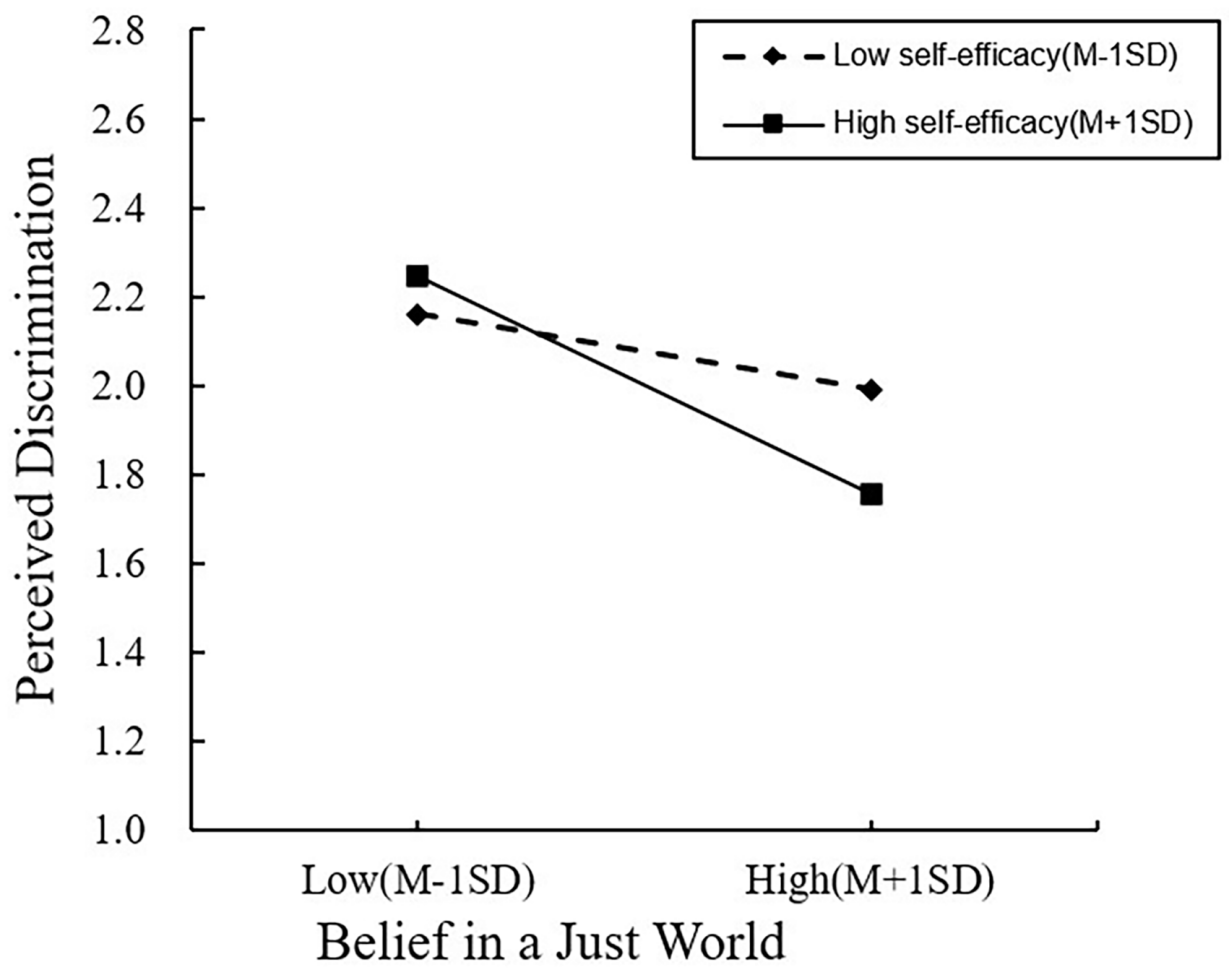
The moderating effect of self-efficacy in the relationship between BJW and PD.

Further, self-efficacy was divided into high and low levels according to M ± 1SD for a simple slope analysis (Figure 2). The results showed that when the level of self-efficacy was higher, the negatively predictive effect of BJW on PD was higher (*β* simple = −0.29, *t* = −5.01, *p* < 0.001, 95% CI = [−0.41, −0.18]). When the level of self-efficacy was lower, the negatively predictive effect of BJW on PD was lower (*β* simple = −0.10, *t* = −2.1, *p* < 0.05, 95% CI = [−0.19, −0.01]). There was a significant difference in indirect effect between the high and low levels (*β* = −0.19, 95% CI = [−0.20, −0.01], *p* < 0.001). Therefore, according to these findings, Hypothesis 2 was supported.

## 5 Discussion

We tested a moderated mediation model to elucidate how BJW relates to LB among Chinese junior high school students. Our research includes three aspects. First, the hypothesis that BJW is negatively correlated with PD is verified. Consistent with SCM theory (Tomaka and Blascovich, 1994), junior high school students with a lower level of BJW appeared to have a higher level of LB.

Second, our findings support Hypothesis 1; BJW negatively predicted LB through reduced PD. Although earlier work focused on direct BJW-LB links (Wang and Meng, 2015; Yu et al., 2023; Zhou and Guo, 2013), we uniquely identified PD mediation among adolescents. This aligns with the stress coping model SCM (Tomaka and Blascovich, 1994), wherein low BJW heightens PD perception, subsequently increasing LB risk. Notably, PD only partially mediated this relationship. Alternative pathways, such as coping styles (Yu et al., 2023), may operate concurrently.

Third, our findings supported Hypothesis 2 that self-efficacy moderates the indirect path from BJW to LB. Specifically, the moderating effect of self-efficacy is manifested in the first path of mediated links (BJW → PD). For junior high school students with low self-efficacy, a low level of BJW appeared to be a risk factor that increases their PD, which in turn increases the likelihood of LB. In addition, for junior high school students with high self-efficacy, the indirect association between a high level of BJW and LB via PD was significantly lower, suggesting that self-efficacy acts as a protective factor that weakens the relationship between risk factors and LB (Chen et al., 2022).

This study establishes a moderated mediation model, where BJW reduces LB via decreased PD, with self-efficacy buffering this pathway. Schools should monitor BJW and self-efficacy levels, implementing evidence-based interventions that simultaneously address environmental (BJW) and individual (self-efficacy) factors to mitigate adolescent LB.

Our post-hoc analyses revealed notable gender and urban–rural disparities. Girls’ elevated PD aligns with prior evidence that adolescent girls are more sensitive to interpersonal inequities and academic stressors. Meanwhile, rural students’ higher PD may reflect systemic inequities: Rural schools often lack resources to counter socioeconomic disadvantages, and cultural stigma may amplify perceived discrimination. These findings underscore the need for tailored interventions for female students’ cognitive–behavioral training to reframe discriminatory experiences. For rural students, school-based programs reduce PD levels through peer mentoring and justice-awareness campaigns. Although demographic variables were not our primary focus, these exploratory results highlight how contextual factors may modulate the BJW–LB pathway, extending the ecological validity of our moderated mediation model.

However, there are still some deficiencies and limitations in this study that need further improvement. First, the measures of BJW, PD, self-efficacy, and LB in junior high school students were all self-assessed through student self-reports, which may introduce reporting biases, and future research would benefit from a multi-method, multi-informant approach incorporating teacher evaluations and parent reports. Second, the cross-sectional design precludes definitive causal claims about the observed relationships. While our moderated mediation model is theoretically grounded, the temporal sequence between BJW, PD, self-efficacy, and LB remains inferential, and future research requires longitudinal examination. Third, while our large sample (*N* = 797) enhances statistical power, all participants were recruited from Shandong Province, China—a region with distinctive educational and cultural characteristics (e.g., Confucian values). This geographic specificity raises important questions about the generalizability of our moderated mediation model to other contexts. Finally, our findings are developmentally bound to junior high school students, and they are not necessarily generalizable outside this student group. Future studies could recruit samples from high school or college students to increase generalizability.

Notwithstanding these limitations, we establish the first evidence-based model demonstrating that BJW mitigates LB through reduced PD, with self-efficacy serving as a protective amplifier. The findings offer actionable intervention targets of LB and provide a testable framework for adaptation in other settings.

Our research also suggests that, in light of the cognitive characteristics of junior high school students, teachers can enhance their BJW proficiency by designing “light task” social scenarios (group cooperation in class) and digital tools (synchronization in parent groups). Previous studies have shown that intervention with BJW reduced the level of LB in individuals (Yu et al., 2023; Zhou and Guo, 2013). To reduce the PD level of junior high school students, the school has achieved the goal of lowering the PD level by regularly running projects, such as “Fair Classroom” role-playing and “Frustration Reconstruction” cognitive training, and visualizing PD through externalization techniques. BJW has a bicultural function, emphasizing group harmony in collectivist culture and the protection of individual rights in individualist culture. In different cultural environments, various approaches can be adopted to enhance the BJW level. In a collectivist culture, the Confucian thought of “self-cultivation and family management” can be utilized to design a collaborative BJW improvement approach between home and school, such as a family ethics drama discussion meeting. In an individualistic culture, critical thinking training can be combined to deconstruct PD through mock court debates. In addition, our study revealed the mediating role of PD and the moderating role of self-efficacy between BJW and LB. These findings suggest that school staff (teachers and psychology teachers) and parents should also monitor the levels of BJW and self-efficacy in junior high school students and raise them through evidence-based practice as needed. In conclusion, developing and implementing intervention programs that include components that address both the environment (BJW) and individual factors (PD and self-efficacy) appear to be key to most effectively dealing with LB in junior high school students.

Although we controlled for age and gender, our initial design did not prioritize demographic comparisons. Thanks to the feedback from the reviewers, we conducted an exploratory group analysis and discovered meaningful changes related to gender and urban–rural background. Future longitudinal studies should explicitly investigate how these factors interact with BJW and self-efficacy over time.

Finally, this research was only conducted in Shandong Province, China. This region is the birthplace of Confucianism and is deeply influenced by Confucian values, emphasizing collective welfare, educational diligence, and hierarchical social harmony. First, in this study, the association between BJW and LB may have been strengthened. The Confucian ideological viewpoints, such as “diligence makes up for deficiency,” can enhance the protective effect of BJW on LB by treating academic effort as a moral requirement. Second, in Confucian culture, BJW places more emphasis on “collective justice” rather than “individual justice” in the West. Self-efficacy may stem more from evaluations by families or teachers than from individual initiative. Therefore, the inherent vertical collectivism of Confucianism may exacerbate PD among rural students, who often attribute educational inequality to systemic bias. Third, we have not quantitatively measured the cultural identity of Confucian culture. Therefore, we are unable to distinguish whether the moderated mediation model reflects a universal psychological mechanism or a specific outcome in a particular culture.

Future cross-cultural research can be considered from the three aspects. First, compare regions with different degrees of influence of Confucian culture, such as Shandong Province and Guangdong Province. Second, a hybrid approach is adopted, such as integrating ethnographic research with quantitative research. Third, this model is tested in an individualistic society where self-efficacy may rely more on individual initiative rather than environmental justice.

## Data Availability

The datasets presented in this study can be found in online repositories. The names of the repository/repositories and accession number(s) can be found at: [Science Data Bank] [doi: 10.57760/sciencedb.17908].
